# Supplementary material for: Underreporting of SARS-CoV-2 infections during the first wave of the 2020 COVID-19 epidemic in Finland—Bayesian inference based on a series of serological surveys
Source: PLoS One. 2023 Jun 23;18(6):e0282094. doi: 10.1371/journal.pone.0282094 (PMC10289354; doi:10.1371/journal.pone.0282094)
Supplement: S1 Table — (PDF) [file pone.0282094.s001.pdf]

Table S1: Parameters of the prior distribution (hyperparameters) in the Estimation model, and the specificities of the screening and confirmation tests.

Parameter	Description	Value
μ_1	Prior expectation for $logit(\pi_1^{(0)})$	logit(0.05)
σ_1	Prior standard deviation for $logit(\pi_1^{(0)})$	2
α	Shape parameter for gamma prior distribution of σ	2
β	Rate parameter for gamma prior distribution of σ	40
δ^{Screen}	Screening test specificity	0.9759
$\delta^{Confirmation}$	Confirmation test specificity	1
