# Supplementary material for: Underreporting of SARS-CoV-2 infections during the first wave of the 2020 COVID-19 epidemic in Finland—Bayesian inference based on a series of serological surveys
Source: PLoS One. 2023 Jun 23;18(6):e0282094. doi: 10.1371/journal.pone.0282094 (PMC10289354; doi:10.1371/journal.pone.0282094)
Supplement: S2 Table — Shown are posterior means and 95% credible intervals for Δ(t), based on the confirmation test data, for 9th April 2020 (t = 0), 28th May 2020 (t = 49) and 2nd July 2020 (t = 84), using different values for the parameters μ1, σ1, and β. The value used for the parameter α was 2. (PDF) [file pone.0282094.s002.pdf]

Table S2: Influence of choices of hyperparameters on the estimation of underreporting ratio $\Delta(t)$. Shown are posterior means and 95% credible intervals for $\Delta(t)$, based on the confirmation test data, for 9th April 2020 ($t = 0$), 28th May 2020 ($t = 49$) and 2nd July 2020 ($t = 84$), using different values for the hyperparameters μ_1, σ_1, and β. The value used for the hyperparameter α was 2.

Hyperparameters			Underreporting ratios		
$logit(\mu_1)$	σ_1	β	$\Delta(t = 0)$	$\Delta(t = 49)$	$\Delta(t = 84)$
0.005	2	2	9.31 (2.571–23.33)	2.49 (0.355– 7.30)	4.61 (0.096–23.61)
0.005	2	20	8.17 (3.15–16.05)	2.48 (0.89– 5.43)	3.69 (0.78–14.05)
0.005	2	40	8.26 (3.27–15.76)	2.40 (0.91– 4.89)	3.04 (0.84– 9.98)
0.005	2	120	8.48 (3.49–15.90)	2.25 (0.92– 4.31)	2.22 (0.82– 5.19)
0.005	10	2	9.36 (2.292–24.94)	2.45 (0.348– 7.13)	4.53 (0.087–23.05)
0.005	10	20	8.13 (2.97–16.08)	2.46 (0.84– 5.40)	3.67 (0.75–13.56)
0.005	10	40	8.28 (3.14–15.76)	2.41 (0.90– 4.93)	3.07 (0.81–10.09)
0.005	10	120	8.36 (3.28–15.87)	2.21 (0.86– 4.31)	2.17 (0.76– 5.01)
0.050	2	2	11.16 (3.175–28.88)	2.54 (0.352– 7.32)	4.61 (0.086–23.02)
0.050	2	20	8.97 (3.54–17.26)	2.61 (0.94– 5.58)	3.82 (0.83–14.01)
0.050	2	40	8.95 (3.75–16.50)	2.56 (1.03– 5.08)	3.15 (0.93– 9.78)
0.050	2	120	9.22 (3.89–17.02)	2.43 (1.01– 4.55)	2.38 (0.89– 5.36)
0.050	10	2	9.47 (2.382–24.46)	2.49 (0.362– 7.16)	4.60 (0.088–22.81)
0.050	10	20	8.18 (3.00–16.30)	2.47 (0.85– 5.37)	3.68 (0.75–13.80)
0.050	10	40	8.22 (3.20–15.88)	2.38 (0.90– 4.87)	2.99 (0.83– 9.64)
0.050	10	120	8.44 (3.37–16.06)	2.23 (0.88– 4.34)	2.21 (0.78– 5.21)
