# Supplementary figures and images for: Underreporting of SARS-CoV-2 infections during the first wave of the 2020 COVID-19 epidemic in Finland—Bayesian inference based on a series of serological surveys
Source: PLoS One. 2023 Jun 23;18(6):e0282094. doi: 10.1371/journal.pone.0282094 (PMC10289354; doi:10.1371/journal.pone.0282094)

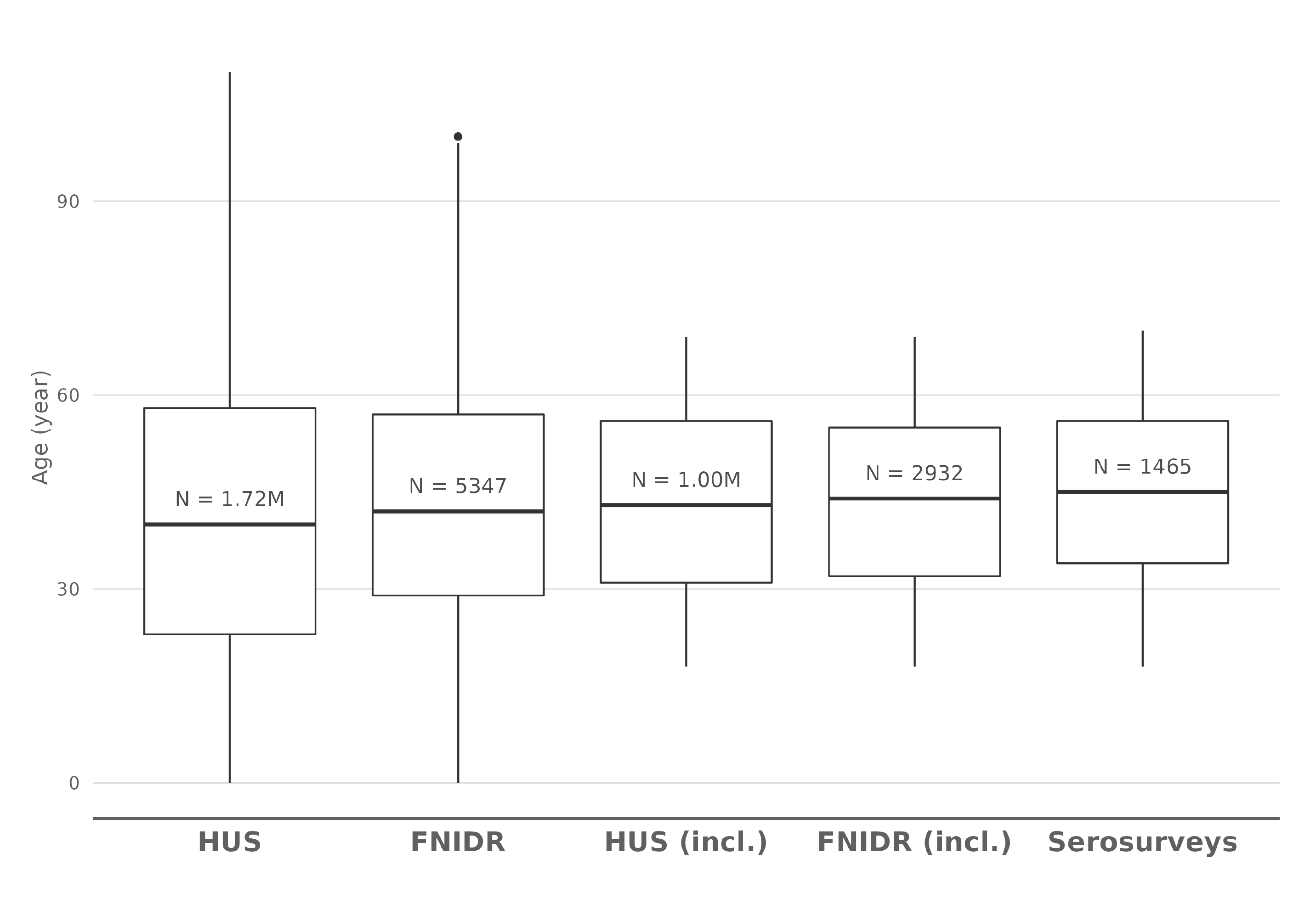

Supplement: S1 Fig — Age distributions of: population in the Helsinki-Uusimaa region at the end of 2021 (HUS); COVID-19 cases for the HUS population during the first wave of the COVID-19 epidemic in 2020 (FNIDR); the study population, i.e. the target population of the current study (HUS (incl.)); COVID-19 cases from the study population during the first wave of the COVID-19 epidemic in 2020 (FNIDR (incl.)); serological survey participants from the study population during the first wave (Serosurveys). (TIF) [file pone.0282094.s003.tif]

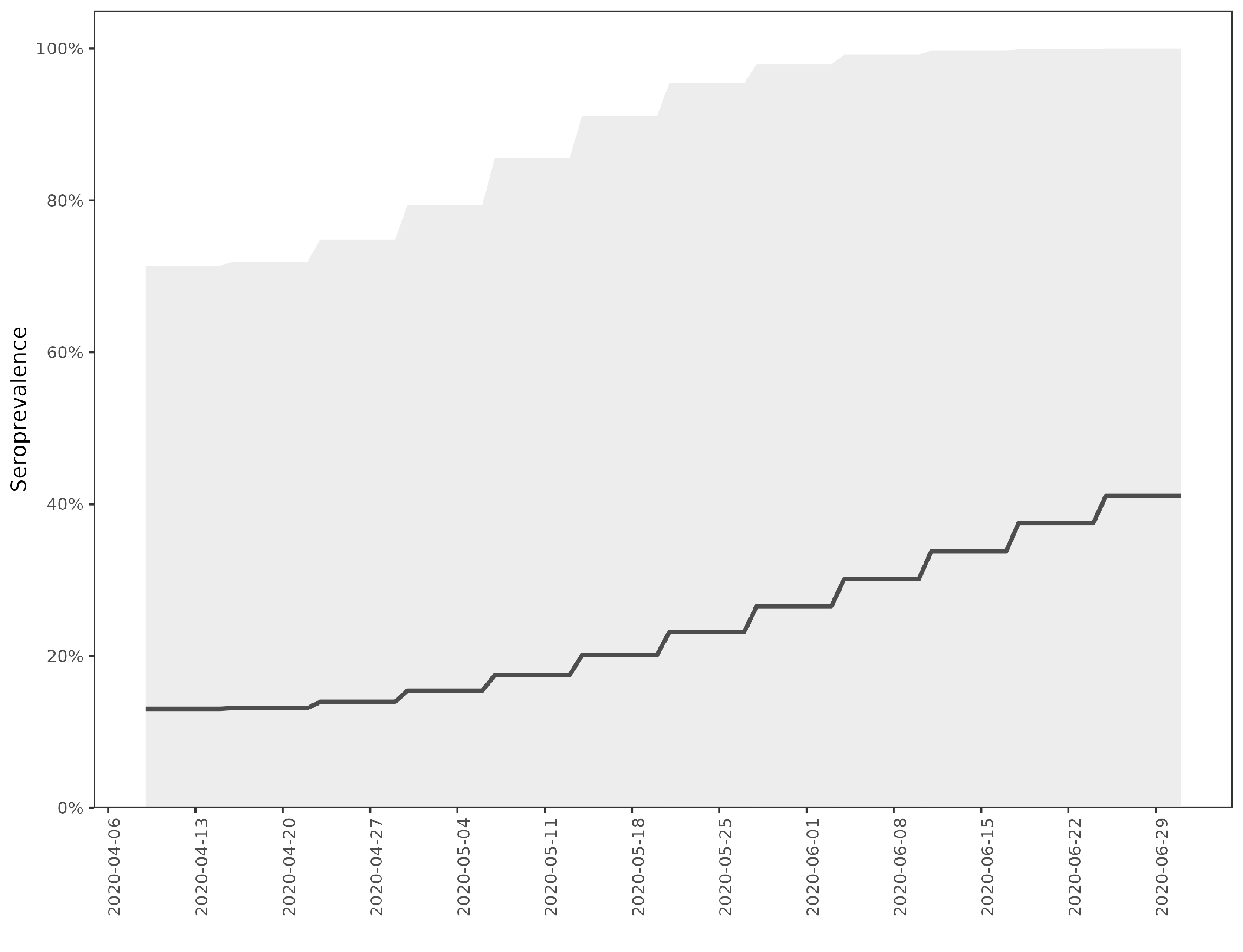

Supplement: S3 Fig — Prior mean, and 2.5% and 97.5% quantiles for each weekly seroprevalence πw(0) in the Estimation model. The estimates were computed based on 40000 samples generated from the prior distribution of π. (TIF) [file pone.0282094.s005.tif]

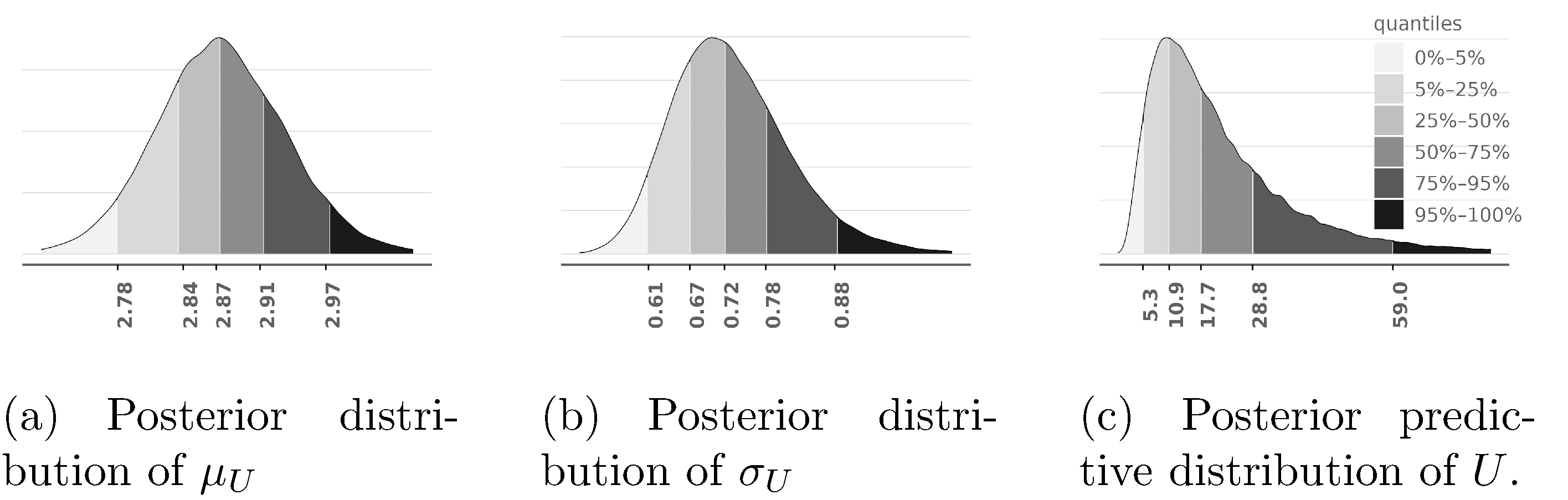

Supplement: S4 Fig — The three images show, starting from the the left: the posterior distribution for μU, the posterior distribution for σU, and the posterior predictive distribution for U, the time from COVID-19 symptom onset to seroconversion. The distribution for U was obtained by sampling from the lognormal distribution, using samples from the joint posterior distribution for (μU, σU). (TIF) [file pone.0282094.s006.tif]

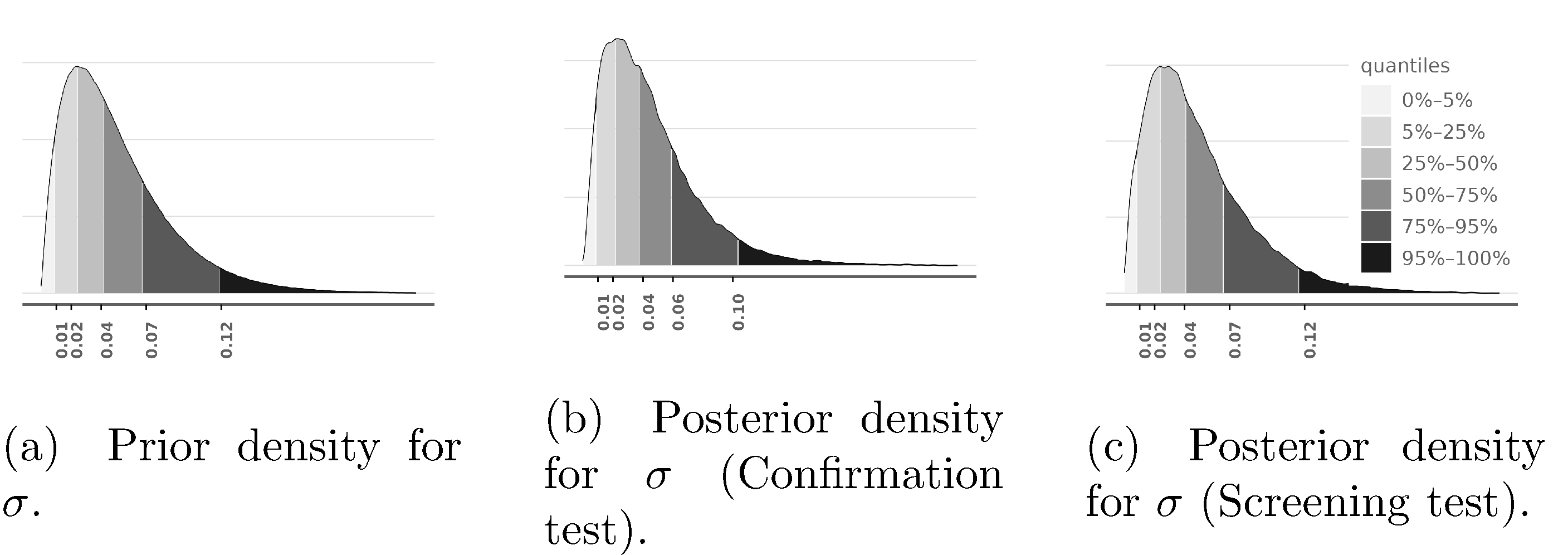

Supplement: S5 Fig — Image on the left shows the prior distribution, the middle image shows the posterior distribution based on confirmation test data, and the image on the right shows the posterior distribution based on the screening test data. (TIF) [file pone.0282094.s007.tif]

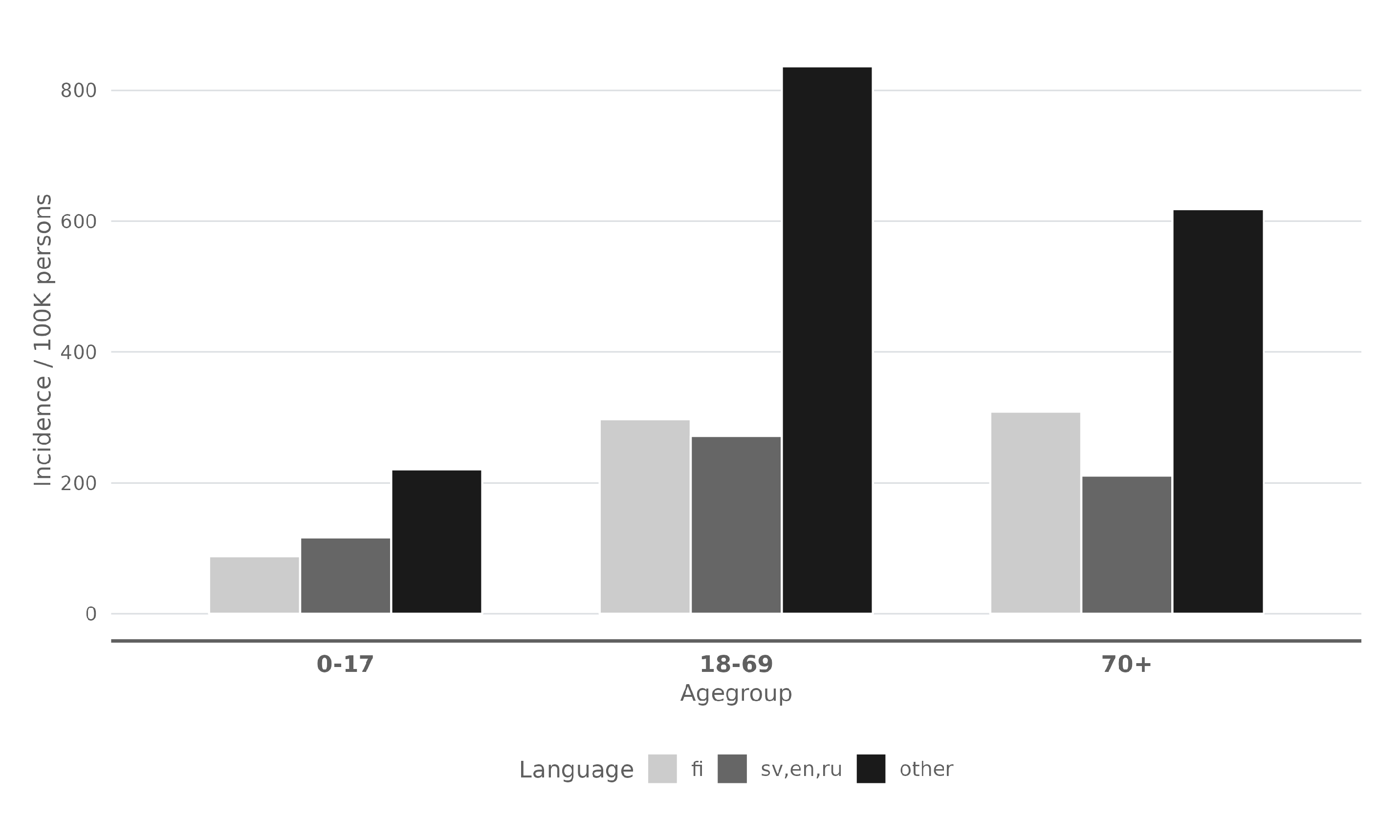

Supplement: S6 Fig — The language groups are Finnish (fi), Swedish (sv), English (en), Russian (ru) and other. (TIF) [file pone.0282094.s008.tif]

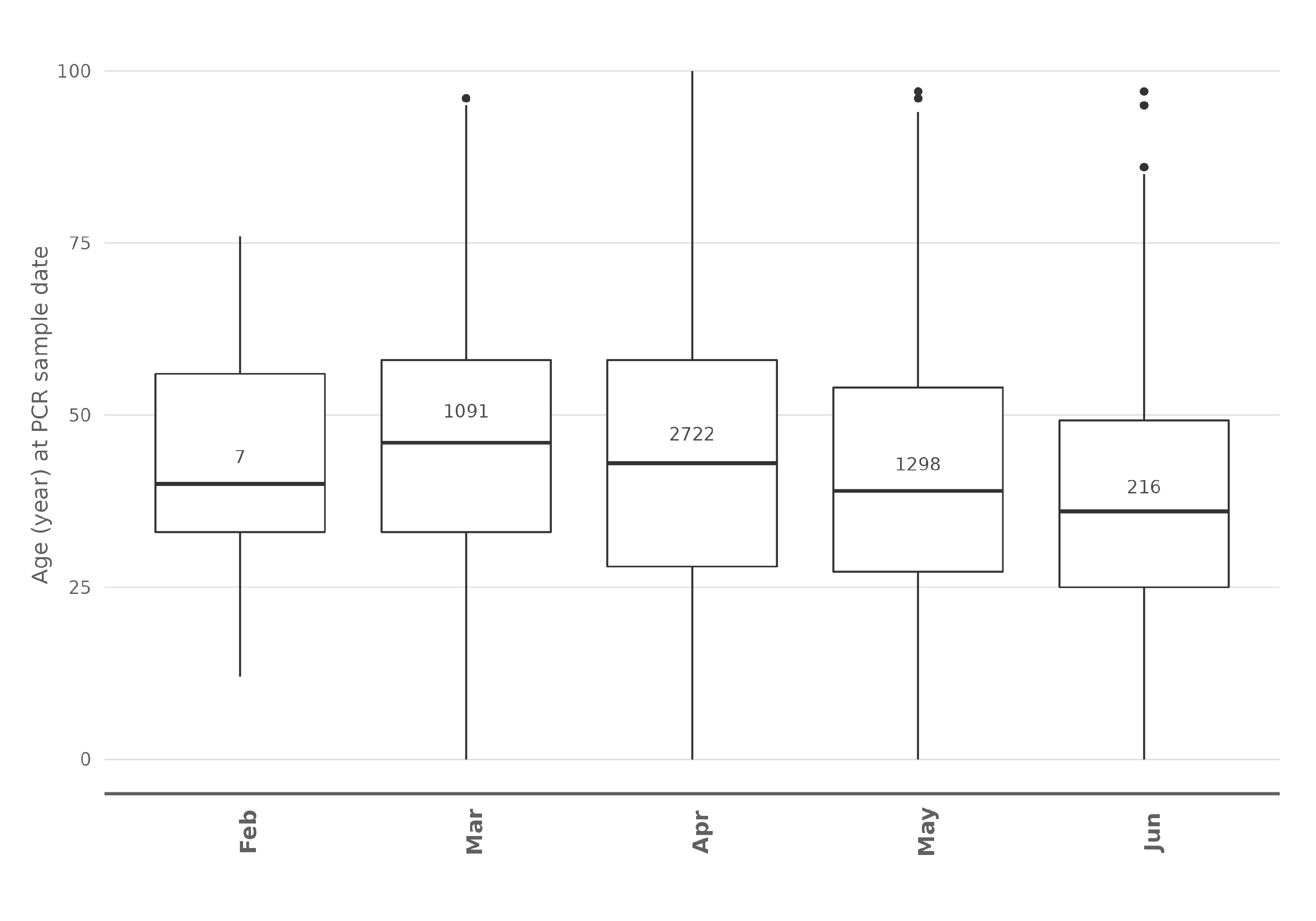

Supplement: S7 Fig — (TIF) [file pone.0282094.s009.tif]
